# Efficacy of Acupuncture, Intravenous Lidocaine, and Diet in the Management of Patients with Fibromyalgia: A Systematic Review and Network Meta-Analysis

**DOI:** 10.3390/healthcare10071176

**Published:** 2022-06-23

**Authors:** Nawaf Masaad Almutairi, Faisal Mohammed Hilal, Ahmed Bashawyah, Fatma Al Dammas, Ece Yamak Altinpulluk, Jin-De Hou, Jui-An Lin, Giustino Varrassi, Ke-Vin Chang, Abdallah El-Sayed Allam

**Affiliations:** 1 Department of Anesthesia and Pain Management, Security Forces Hospital, Riyadh 11481, Saudi Arabia; dr.nawaf@yahoo.com; 2 Saudi Board of Anesthesia SPA, Ministry of Health, King Fahad General Hospital, Jeddah 23325, Saudi Arabia; dr.faisal.hilal@gmail.com; 3 Department of Anesthesiology, College of Medicine, King Abdulaziz University Hospital, Jeddah 22252, Saudi Arabia; dr.bashaweeh@gmail.com; 4 Department of Anesthesia and Pain Management, King Khalid University Hospital and Medical City, Riyadh 61421, Saudi Arabia; fdammas@yahoo.com; 5 MoMaRC Morphological Madrid Research Center, 28029 Madrid, Spain; eceyamak@gmail.com (E.Y.A.); dr_3llam2007@hotmail.com (A.E.-S.A.); 6 Department of Outcomes Research Consortium, Cleveland Clinic, Cleveland, OH 44195, USA; 7 Anesthesiology Clinical Research Office, Ataturk University, 25030 Erzurum, Turkey; 8 Division of Anesthesiology, Hualien Armed Forces General Hospital, Hualien 97144, Taiwan; jindehou805@gmail.com; 9 Department of Anesthesiology, School of Medicine, National Defense Medical Center, Taipei 11490, Taiwan; 10 Center for Regional Anesthesia and Pain Medicine, Wan Fang Hospital, Taipei Medical University, Taipei 116, Taiwan; kvchang011@gmail.com; 11 Department of Anesthesiology, School of Medicine, College of Medicine, Taipei Medical University, Taipei 110, Taiwan; 12 Pain Research Center, Wan Fang Hospital, Taipei Medical University, Taipei 116, Taiwan; 13 Department of Anesthesiology, Wan Fang Hospital, Taipei Medical University, No. 111, Sec. 3, Xinglong Rd., Wenshan Dist., Taipei City 116, Taiwan; 14 Department of Anesthesiology, School of Medicine, Chung Shan Medical University, Taichung 40201, Taiwan; 15 Department of Anesthesiology, Chung Shan Medical University Hospital, Taichung 40201, Taiwan; 16 Paolo Procacci Foundation, 00193 Roma, Italy; giuvarr@gmail.com; 17 Department of Physical Medicine and Rehabilitation, National Taiwan University Hospital, Bei-Hu Branch 10845, Taiwan; 18 Department of Physical Medicine, Rheumatology and Rehabilitation, Faculty of Medicine, Tanta University, Tanta 31527, Egypt

**Keywords:** acupuncture, diet, fibromyalgia, lidocaine, network meta-analysis

## Abstract

**Introduction:** This network meta-analysis aimed to assess the efficacy of acupuncture, intravenous lidocaine, and diet compared with other comparators such as physiotherapy and sham/placebo in fibromyalgia patients. **Materials and Methods:** We searched Embase, PubMed, Scopus, and Web of Science for relevant studies till September 2021. The included studies were randomized controlled clinical trials. For the network meta-analysis, we used the R software. **Results:** There were 23 included RCTs. The total sample size was 1409 patients. Compared with the sham/placebo group, the network analysis showed the highest improvement in the quality of life in the acupuncture group standardized mean difference (SMD) = −10.28, 95%-CI [−14.96; −5.59]), and then in the physiotherapy group (SMD = −7.48, 95%-CI [−14.72; −0.23]). For the pain, there was a significant reduction with acupuncture (SMD = −1.69, 95%-CI [−2.48; −0.89]), compared with sham/placebo. Regarding depression, it showed a significant reduction with acupuncture (SMD = −9.64, 95%-CI [−16.13; −3.14]) compared with sham/placebo. Finally, for stiffness, it showed no significant differences in the stiffness between acupuncture (SMD = −8.52, 95%-CI [−20.40; 3.36]), fluoxetine (SMD = −6.52, 95%-CI [−29.65; 16.61]), and physiotherapy (SMD = −4.64, 95%-CI [−22.83; 13.54]) compared with sham/placebo. **Conclusions:** The acupuncture showed a significant effect in the management of fibromyalgia patients. It reduced pain, depression, and enhanced the quality of life. While physiotherapy showed a significant improvement in the quality of life only. In contrast, intravenous lidocaine and diet showed no significant differences when compared with sham/placebo.

## 1. Introduction

Fibromyalgia (FM) is a condition known for its chronic widespread musculoskeletal pain. It is diagnosed by the American College of Rheumatology (ACR) criteria 2016 [1]. Fibromyalgia patients are usually characterized by moderate-to-severe symptoms including widespread pain, point tenderness, cognitive disturbance, sleep disturbance, psychiatric, and multiple somatic symptoms [1,2,3]. The balance impairments and functionality can considerably influence the FM patients’ quality of life (QoL) [4,5,6]. So, FM patients have difficulties in their daily activities’ performance, and their ability to work [7,8,9,10].

One of the recommended management options for FM includes acupuncture [11]. The reviews offer positive evidence for its efficacy in the improvement of pain and stiffness compared with conventional treatment, or no treatment or drug therapy [12,13]. However, there is no support for its use for physical function disabilities. Acupuncture’s benefits in balance impairment were observed in patients with conditions other than FM [14,15].

FM patients are often treatment-resistant or suffer from unbearable side effects using conventional oral medications. Intravenous (IV) lidocaine is a treatment having peripheral and central-mediated analgesic, anti-inflammatory, as well as antihyperalgesic effects [16,17]. IV lidocaine has been reported to be safe and effective in producing clinically efficient analgesia in patients suffering from various pain disorders [18]. 

In addition to this, the role of diet has also been investigated as a treatment for FM, as there have been various linked deficiencies to FM. However, in spite of the encouraging results, this cannot be solid evidence, as much of the available regarding this topic had poor quality, different study designs, inadequate sample sizes, and absence of control groups [19].

A recent study suggested that FM was significantly associated with higher risks for suicidal ideations, suicide attempts, and death by suicide in comparison with the general population [20]. So, it is crucial to investigate the treatment options for FM patients, whether physically or psychologically related. There is a significant gap in the literature among FM patients treated with acupuncture, lidocaine, and diet. Some studies reported that these interventions were effective, while others reported they were non-effective.

Therefore, the objective of this study was to conduct a network meta-analysis to compare acupuncture, lidocaine, and diet with other comparators such as physiotherapy and sham/placebo in FM patients.

## 2. Materials and Methods

### 2.1. Study Design

This is a systematic review and network meta-analysis, which was performed strictly according to the preferred reporting items of PRISMA guidelines [21,22]. The network meta-analysis is a type of meta-analysis that compares multiple treatments (three or more) by employing direct comparisons of interventions within randomized controlled trials (RCTs) as well as indirect comparisons across trials based on a common comparator. The direct and indirect evidence can be combined as a weighted average, and made comparisons between each arm included.

### 2.2. Literature Search

A systematic search was conducted on Embase, PubMed, Scopus, and Web of Science, till September 2021. We gathered the search terms from MeSH and common search terms used in the related publications. The keywords included Fibromyalgia, Fibromyalgias, Fibromyalgia–Fibromyositis, Muscular Rheumatism, Fibrositis, Fibrositides, Diffuse Myofascial Pain Syndrome, Fibromyositis–Fibromyalgia Syndrome, Fibromyositis Fibromyalgia Syndrome, Lignocaine, Octocaine, Xylesthesin, Xylocaine, Xylocitin, Dalcaine, Diet, Dietary, Acupuncture, Acupanctom, Pharmacoacupuncture, Acupotomy, and Acupotomies. The full-search strategy is presented in Appendix A.

### 2.3. Eligibility Criteria and Study Selection

We included RCTs comparing acupuncture, diet, and lidocaine with other comparators such as physiotherapy and sham/placebo in fibromyalgia patients. All studies included were compared with each other by direct or indirect comparisons. We excluded abstracts, non-English studies, articles other than RCTs, and studies without an outcome of interest or used different scales. To exclude the duplicates, we used Endnote software. Two authors initially screened the title and abstract; then, the other two authors screened the relevant full texts and did a manual screening to ensure all suitable articles were included.

### 2.4. Data Extraction

Three authors extracted data related to the following: (1) Summary and baseline: Study ID, Country, Design, Diagnostic criteria, Study duration (month), Interventions, Sample size, Age, female, Weight, BMI, Years of diagnosis, Fibromyalgia Impact Questionnaire (FIQ), BDI, VAS (mm, mean, SD), Fibromyalgia Severity Scale, and the number of tender points (TPN); (2) The study outcomes: the primary outcome was changed in the QoL, and the secondary outcomes included changes in pain, depression, and stiffness.

### 2.5. Outcomes’ Scales

The scales used for the QoL were the Fibromyalgia Impact Questionnaire (FIQ) [23], and Short Form-36 [24]. For the pain, the used scales were Numeric Rating Scale (NRS) [25], Visual Analogue Scale (VAS) [25], Chronic Pain Grade Scale [25], widespread pain index (WPI) [26], Short Form of the McGill Pain Questionnaire (SF MPQ) [25], and FIQ (pain) [27]. For the depression scales, there were VAS [25], Beck Depression Inventory (BDI) [28], FIQ (depression) [27], Center for Epidemiologic Studies Depression Scale (CES-D) [29], and Hamilton Depression Rating hetero-evaluation Scale (HDRS) [30]. Finally, regarding the stiffness, the used scales were VAS [25], and FIQ (stiffness) [27].

### 2.6. Risk of Bias

We used the Cochrane risk of bias tool for RCTs [31]. The domains include random sequence generation, allocation concealment, blinding of participants and personnel, blinding of outcome assessment, incomplete outcome data, selective reporting, and other bias. The assessment consists of low, high, or unclear risk of bias. Two authors assessed the risk of bias in all studies, and the supervisor solved any disagreements.

### 2.7. Data Synthesis

All analyses were conducted in R software using the net meta package. Data were pooled as standardized mean difference (SMD) (as the outcomes were assessed by different scales) and 95% CI. Data were considered significant if *p* < 0.05. We measured heterogeneity using the I-square test and Chi-Square test. Significant heterogeneity was considered if Chi-Square *p* < 0.1. When heterogeneity was found, we used the random-effect model and tried to solve the heterogeneity by sensitivity analysis using the leave-one-out method. The publication bias was assessed in the outcomes reported in more than 10 studies.

## 3. Results

### 3.1. Literature Search

**Figure 1:**
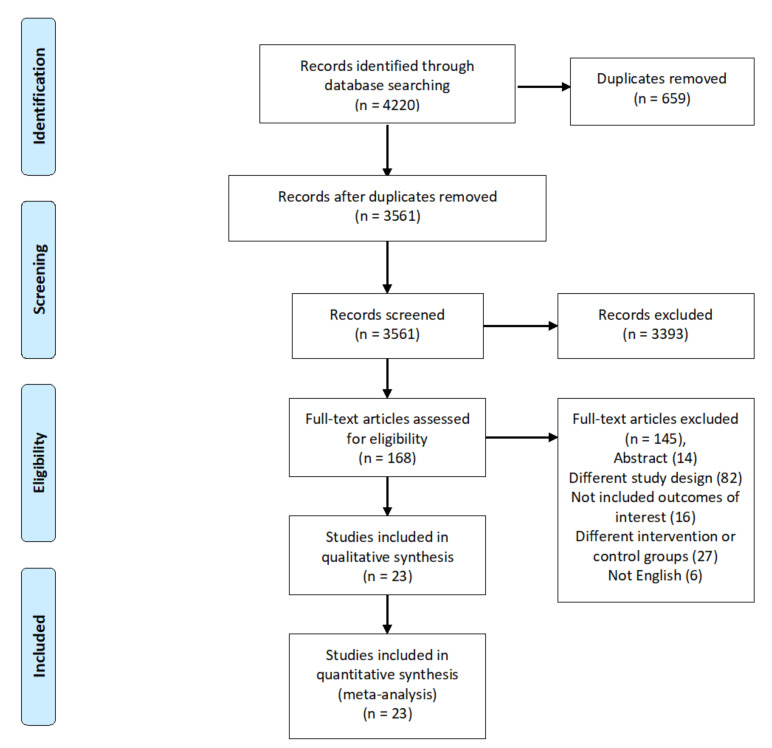
The figure shows the PRISMA flow diagram.

The initially identified records were 4220 in number. Following duplicates’ removal, the remaining records (3561) were screened. Out of them, 168 went through full-text screening. Finally, there were 23 included studies in the analysis [32,33,34,35,36,37,38,39,40,41,42,43,44,45,46,47,48]. Figure 1 PRISMA included the studies’ summary and population’s baseline characters.

All the studies were RCTs. The countries included Spain, Brazil, the USA, Turkey, and others. The interventions included acupuncture, lidocaine, fluoxetine, physiotherapy, nutraceutical, (Inflammatory Gut–Brain Axis Control) (IGUBAC)-Diet, weight reduction, olive tree-based food supplement, transcutaneous electrical nerve stimulation (TENS), cupping, and sham/placebo. The total sample size was 1409 patients. The majority of the included patients were females, see Table 1.

### 3.2. Risk of Bias

**Figure 2:**
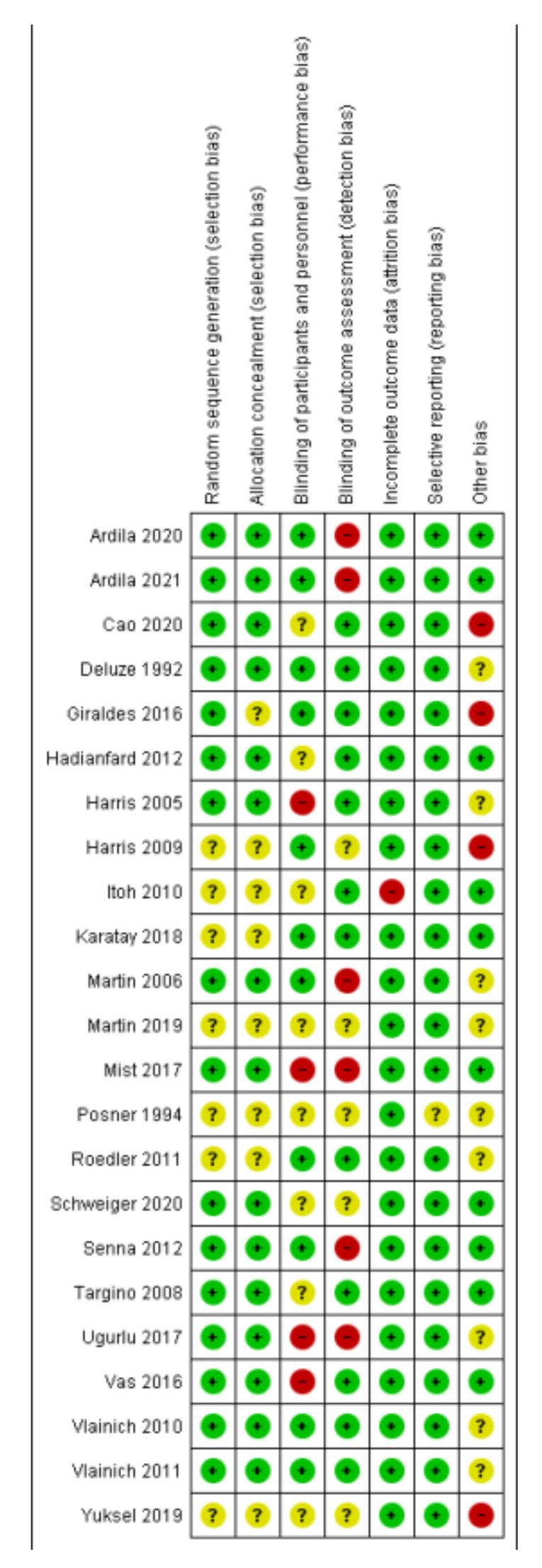
Risk of bias summary.

Most of the included studies had an overall low or moderate risk of bias. However, four of them had a risk of bias as regards the blinding of participants and personnel. In addition, six of them had a high risk of bias regarding the blinding of the outcome assessment. Posner et al. showed an unclear risk of bias for all domains except for the incomplete outcome data, which had a low risk of bias. Other details are provided in Figure 2.

### 3.3. Outcomes

#### 3.3.1. Quality of Life (QoL)

**Figure 3:**
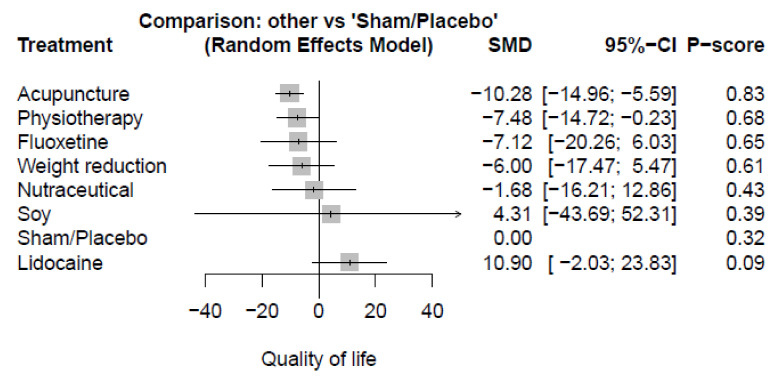
Plot of Quality of life (QoL); SMD: standardized mean difference, CI: confidence interval.

Twelve studies reported the QoL outcome. The pooled estimate of the QoL change from baseline to end-point showed the highest significant improvement in acupuncture (SMD = −10.28, 95%-CI [−14.96; −5.59]) compared with sham/placebo. In addition, the physiotherapy group showed a significant improvement (SMD = −7.48, 95%-CI [−14.72; −0.23]). On the other hand, there were no significant variations between fluoxetine (SMD = −7.12, 95%-CI [−20.26; 6.03]), weight reduction (SMD = −6.00, 95%-CI [−17.47; 5.47]), nutraceutical (SMD = −1.68, 95%-CI [−16.21; 12.86]), soy (SMD = 4.31, 95%-CI [−43.69; 52.31]), and lidocaine (SMD = 10.90, 95%-CI [−2.03; 23.83]) compared with sham/placebo. (Figure 3 and Figure S1) Acupuncture and physiotherapy showed significant variation compared with lidocaine (SMD = −21.18, 95%-CI [−34.92; −7.43], and (SMD = −18.38, 95%-CI [−33.20; −3.56]), respectively. (See Table S1 in the Supplementary File S1 for details).

#### 3.3.2. Pain

**Figure 4:**
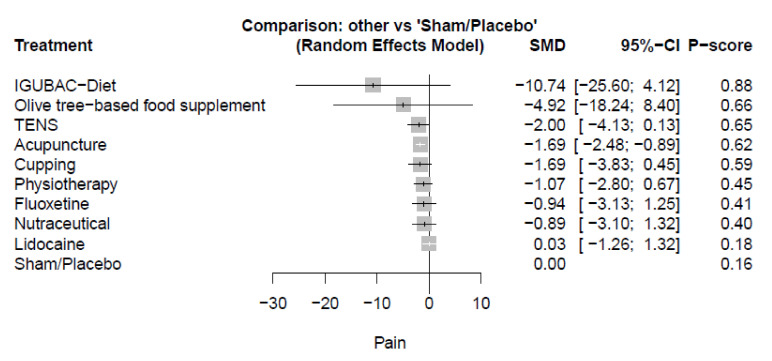
Plot of pain; SMD: standardized mean difference, CI: confidence interval.

Seventeen studies reported the pain outcome. The pooled estimate of pain change from baseline to end-point showed a significant decrease with acupuncture compared with sham/placebo (SMD = −1.69, 95%-CI [−2.48; −0.89]). There were no other significant differences in IGUBAC−Diet (SMD = -10.74, 95%-CI [−25.60; 4.12]), olive tree-based food supplement (SMD = −4.92, 95%-CI [−18.24; 8.40]), TENS (SMD = −2.00, 95%-CI [−4.13; 0.13]), cupping (SMD = −1.69, 95%-CI [−3.83; 0.45]), physiotherapy (SMD = −1.07, 95%-CI [−2.80; 0.67]), fluoxetine (SMD = −0.94, 95%-CI [−3.13; 1.25]), nutraceutical (SMD = −0.89, 95%-CI [−3.10; 1.32]), and lidocaine (SMD = 0.03, 95%-CI [−1.26; 1.32]) compared with sham/placebo. (Figure 4 and Figure S2) Other significant variations were found in acupuncture compared with lidocaine (SMD = −1.72, 95% [−3.23; −0.20]). There were no other significant differences between any measured comparators. (See Table S2 in the Supplementary File S1 for details).

#### 3.3.3. Depression

**Figure 5:**
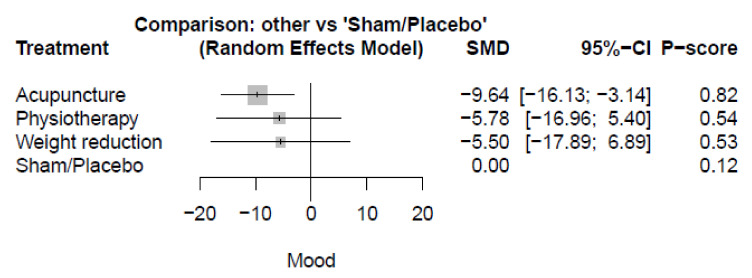
Plot of depression; SMD: standardized mean difference, CI: confidence interval.

Seven studies reported the depression outcome. The acupuncture showed the highest significant reduction in depression (SMD = −9.64, 95%-CI [−16.13; −3.14]) compared with sham/placebo. However, there were no significant differences in physiotherapy (SMD = −5.78, 95%-CI [−16.96; 5.40]), or weight reduction (SMD = −5.50, 95%-CI [−17.89; 6.89]) compared with sham/placebo. (Figure 5 and Figure S3) In addition, there were no other significant differences between the various comparators. (See Table S3 in the Supplementary File S1  for details).

#### 3.3.4. Stiffness

**Figure 6:**
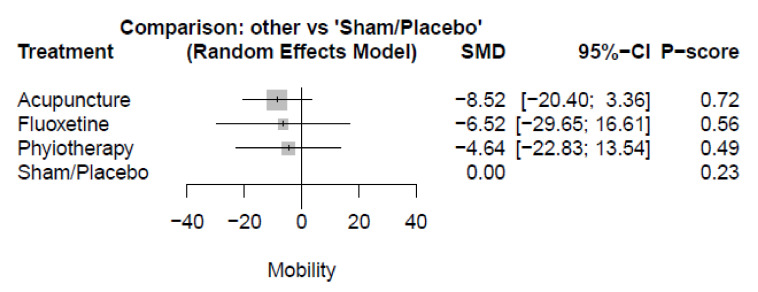
Plot of stiffness; SMD: standardized mean difference, CI: confidence interval.

Four studies assessed the stiffness outcome. The pooled estimate of stiffness change from baseline to end-point showed non-significant differences between acupuncture (SMD = −8.52, 95%-CI [−20.40; 3.36]), fluoxetine (SMD = −6.52, 95%-CI [−29.65; 16.61]), and physiotherapy (SMD = −4.64, 95%-CI [−22.83; 13.54]) compared with sham/placebo. (Figure 6 and Figure S4) There were no other significant differences between the various comparators. (See Table S4 in the Supplementary File S1 for details).

### 3.4. Heterogeneity

All outcomes assessed under random effect model and the results were heterogeneous (*p* < 0.0001, I2 = 95.3–99.4%). The heterogeneity could not be solved by the leave-one-out method.

### 3.5. Publication Bias

**Figure 7:**
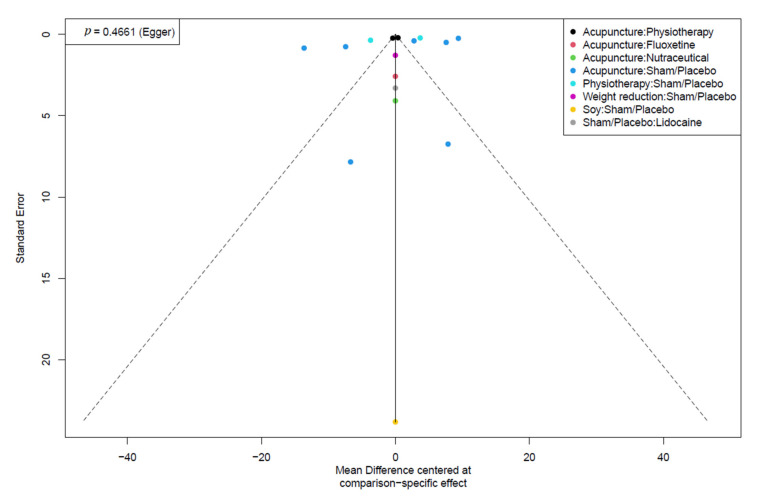
Funnel plot of the QoL outcome.

**Figure 8:**
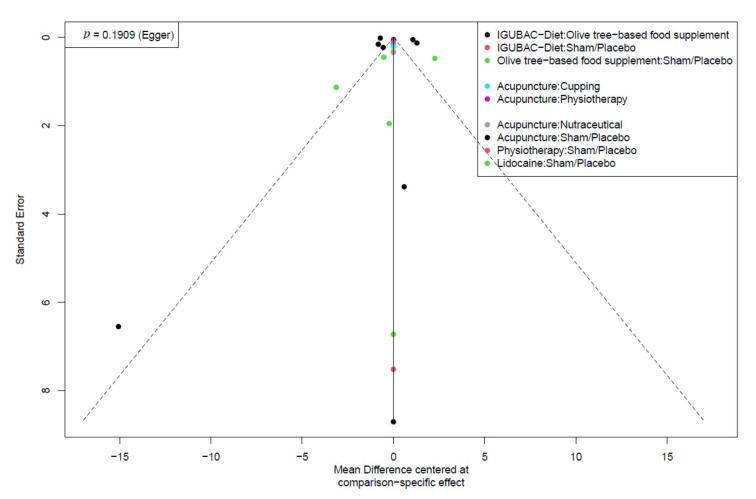
Funnel plot of the pain outcome.

According to Egger et. al., we could not assess the publication bias of the stiffness and depression outcomes because the analyses included less than ten studies. We noticed no publication bias in the QoL and pain outcomes by visual inspection of the funnel plot and *p*-value of Egger’s test. Figure 7 and Figure 8 showed the funnel plots of the QoL and pain outcomes, respectively.

## 4. Discussion

The results showed a significant improvement in the QoL with acupuncture and physiotherapy, and the other comparators showed no significant differences. As regards the pain, the study showed a significant decrease in the acupuncture group only. Regarding depression, there was a significant reduction with acupuncture. On the other hand, there were no significant variations with physiotherapy, or weight reduction. Finally, regarding the stiffness, the results showed no significant differences between acupuncture, fluoxetine, and physiotherapy compared with sham/placebo.

Mayhew and Ernst previously did not recommend acupuncture for FM in their meta-analysis of RCTs [49]. Other reviews also had similar negative conclusions and results to them [13,50,51]. Furthermore, a Cochrane review of 9 studies with 395 patients showed large differences in results. Three showed small, short-lasting effects (all electro-acupuncture), and six did not differ from sham acupuncture [12]. All these reviews included a small number of studies with a small sample size, which may affect the results. In addition, the editorial by Colquhoun and Novella reported that acupuncture is ineffective, and the trials’ positive results are probably false-positive results due to multiple reasons, including bias and low prior probability [52]. On the other hand, the most recent meta-analysis of them concluded that acupuncture was both effective and safe as a treatment for FM patients and recommended its use for FM management [53]. Similar to our study, they also showed pain reduction, and an improvement in the QoL. The analgesic effects of acupuncture activate both the peripheral and central pain control systems through the release of different endogenous opioids or nonopioid compounds, for example, beta-endorphins, enkephalins, dynorphins, serotonin, norepinephrine, and others [54,55,56].

In FM patients, nutritional therapy may be controversial, yet promising. Improvements in pain and functional repercussion in FM patients appeared to occur with a hypo-caloric diet, a raw vegetarian diet, or a diet low in fermentable oligo-, di-, monosaccharides, and polyols, in addition to the QoL and sleep, anxiety and depression, and inflammatory biomarkers. However, those data had several limitations [57]. Various other studies showed promising but limited data about the role of diet in FM patients [58,59]. Our study showed contrary results, as the diet did not show significant differences.

In a previous pilot study, lidocaine showed improvements in the VAS pain scale in FM patients, suggesting that periodic IV infusion of lidocaine might offer extra benefits for FM patients receiving conventional treatment [41]. On the other hand, later on, other studies showed that lidocaine had no improvements when added to amitriptyline [34,60]. Similar to them, our study showed no significant improvements with lidocaine. Giraldes et al. suggested that the absence of difference in the analgesic effect might have been because amitriptyline alone had a sufficient effect. They also suggested that lidocaine required to be administered at smaller intervals to gain a difference in effect [60].

Our inclusion of RCTs only, and the moderate to high quality of the included studies added more validity to our data. Even though our study included various studies, those studies mainly included a small sample size, which limits our data. Another limitation in our study is the different ways of diagnosing fibromyalgia patients in each included study, even when using the ACR criteria. Furthermore, most of the included studies were mainly about acupuncture, and they had different types, points, and numbers of acupuncture. Another limitation includes that some studies had different time points.

Therefore, we recommend future research focus on assessing the effect of diet and lidocaine on FM patients. We also recommend future research have similar time points and use a specific scale for each outcome.

## 5. Conclusions

Acupuncture is a preferred option in fibromyalgia patients compared with other ones investigated in our study, as it showed significant improvements in the quality of life, depression, and pain relief. While physiotherapy showed a significant increase in the quality of life only, when compared with sham/placebo. In addition, lidocaine showed no significant improvements in pain reduction or quality of life when compared with sham/placebo.

## Figures and Tables

**Table 1:** Summary and baseline characteristics of the included studies.

Study ID	Country	Diagnostic Criteria	Duration (Month)	Interventions	Sample Size	Age	Female	Weight	BMI	Years of Diagnosis	FIQ	BDI	VAS (mm, Mean, SD)	Fibromyalgia Severity Scale	Number of Tender Points (TPN)	Acupuncture Site
Ardila 2020	Spain	NR	12	Acupuncture	34	56.15 (7.90)	34 (100)	NR	NR	8.59 (5.18)	4.31 (2.40)	NR	NR	NR	NR	GV20 (Baihui 百会): Highest point in the head, in the mid-point of the line connecting the apexes of the two auricles; Bilateral ST36 (Zusanli 足 三 里): On the anterior aspect of the lower leg, 3cun below the inferior edge of the patella, and 1cun from the anterior crest of the tibia; Bilateral BL60 (Kunlun 昆仑): On the foot, behind the external malleolus, in the depression between the tip of the external malleolus, and the calcaneus tendon.
				Physiotherapy	36	56.06 (8.37)	36 (100)			8.03 (6.30)	4.37 (1.97)					
				Sham	33	54.39 (8.20)	33 (100)			8.30 (4.54)	4.20 (1.81)					
Ardila 2021	Spain	NR	12	Acupuncture	34	56.15 (7.90)	34 (100)	NR	NR	8.59 (5.18)	68.97 (16.98)	NR	7.12 (2.04)	NR	NR	GV20, ST36, and BL60
				Physiotherapy	36	56.06 (8.37)	36 (100)			8.03 (6.30)	70 (17.46)		7.19 (2.1)			
				Sham	33	54.39 (8.20)	33 (100)			8.30 (4.54)	64.42 (15.03)		7.15 (2.06)			
Cao 2020	China	ACR criteria (2010)	8	Randomized acupuncture	30	54.5 (2) *	22 (72.4)	64.6 (11.7)	NR	NR	29.3 (14.8) *	NR	66 (15.9)	NR	NR	Ashi (tender) points
				Randomized cupping	30	53 (16) *	23 (75.9)	64.2 (10.6)			40.2 (20.2) *		73 (14)			
Deluze 1992	Switzerland	ACR criteria (1990)	NR	Acupuncture	36	46.8 (2.3)	3 (8.4)	NR	NR	14.4 (3.7)	NR	NR	56.61 (3.19)	NR	NR	Four common points: the first dorsal interosseous muscle of the hand and the anterior tibial muscle on both sides. Others: Based on the patient’s symptoms and pain and the empirical efficacy of the sites in the treatment of pain
				Sham	34	49 (2)	13 (38.3)			6.9 (1.3)			60.89 (4.07)			
Giraldes 2016	Brazil	ACR criteria (1990)	2	Lidocaine	21	42.4 (9.4)	19 (90.5)	69.8 (13.8)	25 (4.6)	5 (4.2)	65.1 (11.2)	NR	NR	NR	15.2 (2.5)	NA
				Placebo	21	47 (9.8)	21 (100)	65.2 (10.1)	24.2 (3.5)	6 (4.1)	63.5 (15.4)				15.1 (2.9)	
Hadianfard 2012	Iran	ACR criteria (1990)	12	Acupuncture	15	43.86 (7.9)	15 (100)	NR	NR	6.9 (5.7)	38.1 (12.1)	NR	7.5 (1.8)	NR	15.8 (2.1)	ST-36, GB-34, RN-6, SP-6, LI-4, ST-44, BL-40, HT-7, and DU-20
				Fluoxetine	15	44.2 (10.8)	15 (100)			6.6 (5.8)	42.7 (9.6)		7.5 (1.8)		15.5 (2.1)	
Harris 2005	USA	ACR criteria (1990)	3.5	Acupuncture	30	44.5 (10.9)	27 (90)	NR	NR	5.26 (4.83)	NR	NR	NR	NR	NR	Du 20, LI 11, LI 4, GB 34, bi-lateral St 36, Sp 6, Liv 3, and Ear-Shenmen
				Sham	27	48.1 (10.9)	26 (96.3)			5.77 (4.10)						
Harris 2009	USA	ACR criteria (1990)	1	Acupuncture	10	44.3 (13.6)	10	NR	NR	NR	NR	NR	NR	NR	NR	Du20, ear Shenmen, LI4, LI11, Sp6, Liv3, GB34, and bilateral St36
				Sham	10		10									
Itoh 2010	Japan	ACR criteria (1990)	2.3	Acupuncture	6	47.3 (13.3)	NR	NR	NR	4.4 (2.3)	66.3 (11.0)	NR	77.9 (10.1)	NR	NR	Four common acupuncture points, and up to ten additional sites depending on the patient’s symptoms and pain pattern and the empirical choice of trigger point in pain treatment
				Sham	7	45.7 (15.2)				3.9 (3.9)	64.3 (6.4)		74.2 (8.4)			
Karatay 2018	Turkey	ACR criteria (1990)	3	Acupuncture	24	34.7 (6.09)	NR	NR	26.49 (5.65)	4.44 (3.99)	70.8 (12.5)	37.6 (18.8)	8.10 (2.52)	NR	15.9 (2.8)	Du-14 (DaZhui), Si-15 (Jian Zhong Shu), Li-4 (He Gu), Li-11 (QuChi), H-7 (Shen Men), P-6 (Nei Guan), Ren-6 (Qihai), Liv-3 (Tai Chong), St-36 (Zu San Li), and Sp-6 (San Yin Jiao). All were bilateral, apart from Du-14 and Ren-6
				Sham	25	34.2 (6.84)			26.94 (4.63)	3.94 (3.30)	65.8 (24.1)	36.6 (16.7)	7.73 (1.98)		15.9 (2.4)	
Martin 2006	Georgia	ACR criteria (1990)	7	Acupuncture	25	51.7 (14.1)	25 (100)	NR	NR	NR	42.4 (11)	NR	NR	40.4 (10.3)	NR	Bilateral points at large intestine 4, stomach 36, liver 2, spleen 6, pericardium 6, and heart 7. Others: Axial paramedian points along the bladder meridian at the cervical spine during the first 3 sessions
				Sham	24	47.9 (11.2)	23 (95.9)				44 (9.8)			43.0 (7.7)		
Martin 2019	Spain	NR	2	IGUBAC Diet	8	62.1 (7.8)	8 (100)	64.9 (5.9)	26.1 (3.6)	NR	NR	NR	NR	NR	NR	NA
				Placebo	14	55.9 (11.9)	14 (100)	67.7 (10.6)	26.8 (4.16)							
Mist 2017	USA	ACR criteria (1990)	6	Acupuncture	16	52.3 (12.9)	16 (100)	NR	33.2 (10.2)	NR	51.1 (15.9)	NR	6.2 (1.8)	NR	NR	Depends on a combination of TCM Syndrome diagnosis and symptom management
				Sham	14	56 (12.0)	14 (100)		32.7 (7.7)		52.8 (14.0)		6.3 (1.4)			
Posner 1994	Island	ACR criteria (1990)	NR	Lidocaine	11	37.9 (10.9)	11 (100)	NR	NR	NR	NR	NR	7.2 (1.2)	NR	NR	NA
				Placebo	10	32.6 (8.4)	10 (100)						6.2 (1.2)			
Roedler 2011	USA	ACR criteria (1990)	6	Soy	12	47.5 (16.7)	12 (100)	NR	NR	NR	NR	NR	NR	NR	NR	NA
				Placebo	16		16 (100)									
Schweiger 2020	Italy	ACR criteria (2016)	6	acupuncture	34	52.9 (8.5)	34 (100)	NR	NR	NR	74.2 (18.2)	NR	8.5 (1.4)	23.4 (4)	NR	The most recurrent points were: dumai (Governing Vessel) 20 and 24, heart 7, large intestine 4, renmai (Conception Vessel) 12 and 6, gall bladder 21 and 34, stomach 36, spleen 6, bladder 60, kidney 3, and liver 3. The choice of acupoints was personalized, and some of the acupoints were combined
				Nutraceutical	26	48.2 (7.4)	26 (100)				69 (15.9)		7.7 (1.7)	21.5 (5.2)		
Senna 2012	Egypt	ACR criteria (1990)	6	Placebo	42	46.3 (14.4)	38 (90.5)	NR	32.8 (1.4)	8.6 (4)	53.2 (11.6)	17.9 (8.9)	NR	NR	17.2 (1.6)	NA
				Weight reduction	41	44.8 (13.6)	37 (90.2)		32.3 (1.4)	9.8 (4.9)	54.6 (13.1)	18.6 (8.7)			16.2 (2.2)	
Targino 2008	Brazil	ACR criteria (1990)	24	Acupuncture	34	52.09 (10.97)	34 (100)	NR	NR	9.9 (9.7)	NR	NR	7.5 (1.8)	NR	15.8 (2.1)	Ex-HN-3 and bilateral LR3, LI4, PC6, GB34, and SP6 points
				Sham	24	51.17 (11.20)	24 (100)			7.8 (6.3)			7.5 (1.8)		15.5 (2.1)	
Ugurlu 2017	Turkey	ACR criteria (1990)	2	Acupuncture	25	47.28 (7.86)	25 (100)	NR	NR	6.28 (4.97)	60.75 (910.88)	28.24 (8.87)	8.28 (1.45)	55.28 (4.17)	NR	LI 4, ST 36, LV 3, GB 41, GB 34, GB 20, SI 3, SI 4, UB 62, UB 10, SP 6, HT 7, DU 20, DU 14, Kd 27, Ren 6, and PC 6
				Sham	25	43.60 (8.18)	25 (100)			6.32 (2.21)	63.92 (5.43)	28.44 (9.30)	8.60 (1.25)	57.28 (6.27)		
Vas 2016	Spain	ACR criteria (2010)	12	Acupuncture	80	52.3 (9.6)	80 (100)	NR	28.5 (6.4)	5.9 (3.7)	71.7 (11)	NR	79.3 (11)	NR	15.6 (2.4)	NR
				Sham	82	53.2 (9.6)	82 (100)		27.7 (5.4)	5.8 (3.6)	70.1 (14.2)		75.8 (13.3)		15.5 (2.5)	
Vlainich 2010	Brazil	ACR criteria (1990)	1	Lidocaine	15	40.9 (11.6)	15 (100)	NR	25.6 (4.1)	6.8 (5.3)	NR	NR	NR	NR	15.6 (2.4)	NA
				Placebo	15	44.7 (10.5)	15 (100)		28.1 (5.9)	5.6 (3.7)					14.3 (1.8)	
Vlainich 2011	Brazil	ACR criteria (1990)	3	Lidocaine	15	40.9 (11.6)	15 (100)	NR	25.6 (4.1)	NR	NR	NR	NR	NR	NR	NA
				Placebo	15	44.7 (10.5)	15 (100)		28.1 (5.9)							
Yuksel 2019	Turkey	ACR criteria	NR	Acupuncture	21	44.6 ± 10.34	NR	NR	NR	7.8 (5.7)	NR	NR	4.5 (1.8)	NR	NR	Houxi (SI 3), Wangu (SI 4), Shenmai (UB 62), Jinggu (UB 64), Shugu (UB 65), and Yintang
				TENS	21	38.05 ± 11.3				4.25 (2.02)			5.25 (2.6)			

BMI: body mass index, FIQ: fibromyalgia impact questionnaire, BDI: Beck Depression Inventory, VAS: visual analogue scale, SD: standard deviations, NR: not reported, ACR: American College of Rheumatology, IGUBAC: inflammatory gut–brain axis control, TENS: transcutaneous electrical nerve stimulation, cun: width of a person’s thumb at the level of the knuckle, *: median (quartile).

## Data Availability

The datasets generated during and/or analyzed during the current study are available in the Supplementary Files S2, S3, S4, and S5.

## Supplementary Materials

The following supporting information can be downloaded at: https://www.mdpi.com/article/10.3390/healthcare10071176/s1.

## Appendix A. The Full Search Strategy of Databases

#1 = (“Acupuncture” OR “Acupuncture Treatment” OR “Acupuncture Treatments” OR “Treatment, Acupuncture” OR “Therapy, Acupuncture” OR “Pharmacoacupuncture Treatment” OR “Pharmacoacupuncture Therapy” OR “Acupotomy” OR “Acupotomies”)

#2 = (“Diet” OR “Diet Therapies” OR “Therapy, Diet” OR “Restrictive Diet Therapies” OR “Restrictive Diet Therapy” OR “Restriction Diet Therapy” OR “Dietary Restriction” OR “Dietary Restrictions” OR “Restriction, Dietary” OR “Dietary Modification” OR “Dietary Modifications” OR “Modification, Dietary” OR “Diet Modification” OR “Diet Modifications” OR “Modification, Diet”)

#3 = (“Lidocaine” OR “2-(Diethylamino)-N-(2,6-Dimethylphenyl)Acetamide” OR “Lignocaine” OR “Lidocaine Carbonate” OR “Lidocaine Hydrocarbonate” OR “Lidocaine Hydrochloride” OR “Xyloneural” OR “Octocaine” OR “Xylocitin”)

#4 = (“Fibromyalgia” OR “Fibromyalgias” OR “Rheumatism, Muscular” OR “Muscular Rheumatism” OR “Fibrositis” OR “Fibromyalgia, Secondary” OR “Secondary Fibromyalgia” OR “Secondary Fibromyalgias” OR “Fibromyalgia, Primary” OR “Primary Fibromyalgia” OR “Primary Fibromyalgias”)

#5 = (#1 OR #2 OR #3)

#6 = (#4 AND #5)
