# Supplementary material for: Efficacy of Acupuncture, Intravenous Lidocaine, and Diet in the Management of Patients with Fibromyalgia: A Systematic Review and Network Meta-Analysis
Source: Healthcare (Basel). 2022 Jun 23;10(7):1176. doi: 10.3390/healthcare10071176 (PMC9320380; doi:10.3390/healthcare10071176)
Supplement: Supplementary file 1 [file healthcare-10-01176-s001.zip › supplementary material.pdf]

Figure S1. Quality of life (QoL) graph.

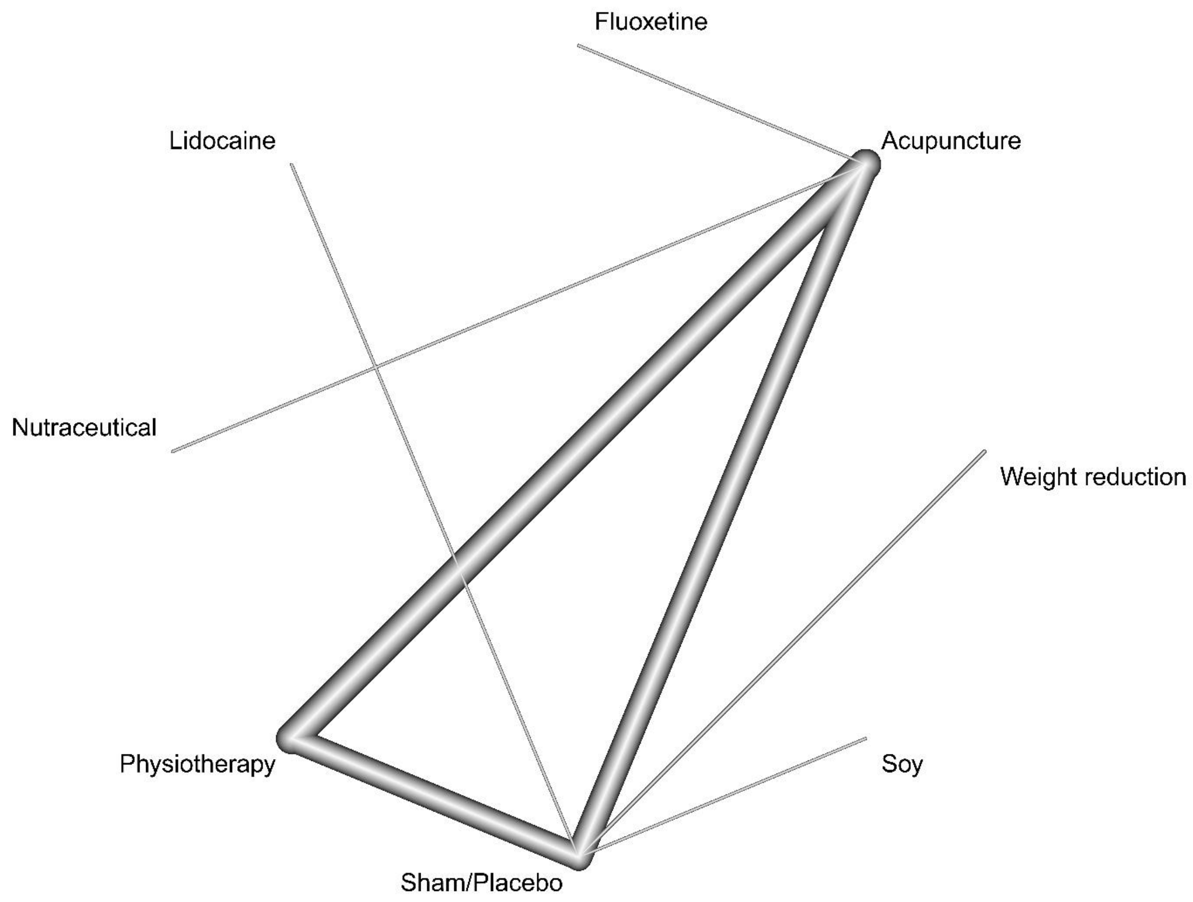

tau-squared = 32.57; I-squared = 99.4%; P < 0.0001

Table S1. A network meta-analysis result regarding quality of life (QoL) change.

Acupuncture							
−2.80 [−10.04; 4.45]	Physiotherapy						
−3.16 [−15.44; 9.12]	−0.36 [−14.62; 13.89]	Fluoxetine					
−4.28 [−16.66; 8.11]	−1.48 [−15.04; 12.09]	−1.12 [−18.56; 16.33]	Weight reduction				
−8.60 [−22.36; 5.16]	−5.80 [−21.35; 9.75]	−5.44 [−23.88; 13.00]	−4.32 [−22.84; 14.19]	Nutraceutical			
−14.59 [−62.82; 33.64]	−11.79 [−60.34; 36.76]	−11.43 [−61.20; 38.34]	−10.31 [−59.66; 39.04]	−5.99 [−56.14; 44.17]	Soy		
**−10.28 [−14.96; −5.59]**	**−7.48 [−14.72; −0.23]**	−7.12 [−20.26; 6.03]	−6.00 [−17.47; 5.47]	−1.68 [−16.21; 12.86]	4.31 [−43.69; 52.31]	Sham/Placebo	
**−21.18 [−34.92; −7.43]**	**−18.38 [−33.20; −3.56]**	−18.02 [−36.45; 0.42]	−16.90 [−34.18; 0.38]	−12.58 [−32.03; 6.87]	−6.59 [−56.30; 43.12]	−10.90 [−23.83; 2.03]	Lidocaine

Figure S2. Pain graph.

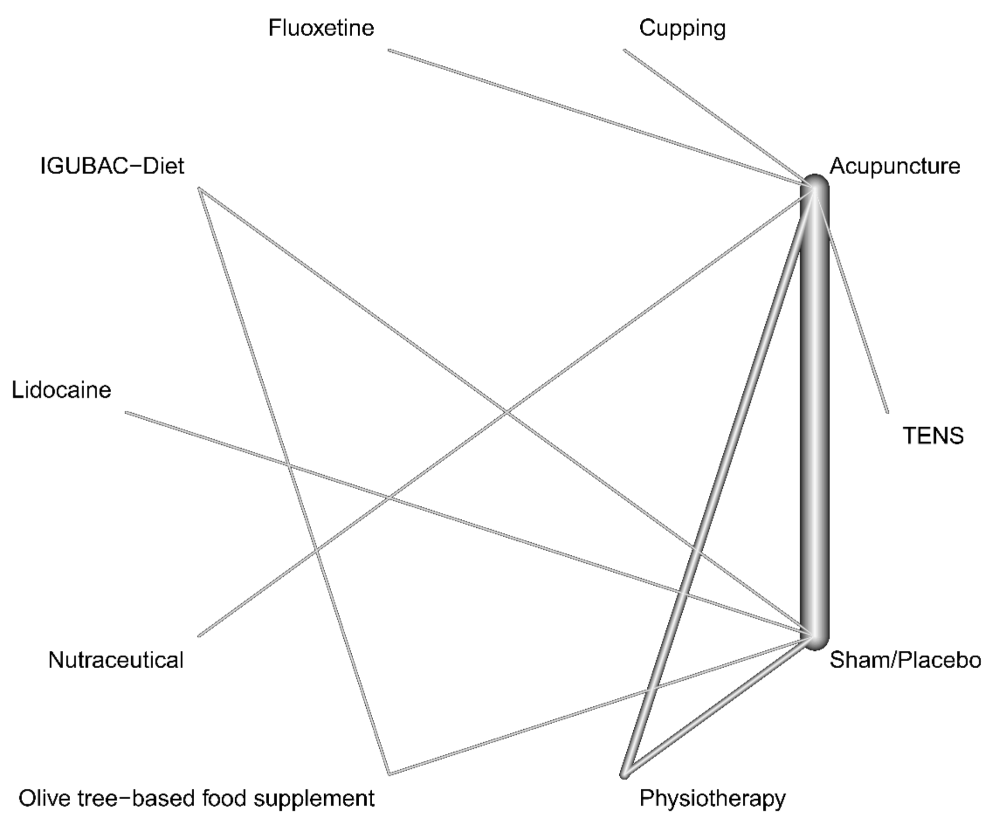

tau-squared = 0.99; I-squared = 99.2%; P < 0.0001

Table S2. A network meta−analysis results regarding pain change.

IGUBAC−Diet									
−5.82 [−22.99; 11.35]	Olive tree−based food supplement								
−8.74 [−23.75; 6.27]	−2.92 [−16.41; 10.57]	TENS							
−9.05 [−23.93; 5.83]	−3.23 [−16.58; 10.11]	−0.31 [−2.28; 1.66]	Acupuncture						
−9.05 [−24.06; 5.96]	−3.23 [−16.72; 10.26]	−0.31 [−3.11; 2.49]	0.00 [−1.99; 1.99]	Cupping					
−9.67 [−24.63; 5.29]	−3.85 [−17.29; 9.58]	−0.93 [−3.56; 1.69]	−0.62 [−2.36; 1.11]	−0.62 [−3.26; 2.02]	Physiotherapy				
−9.80 [−24.82; 5.22]	−3.98 [−17.48; 9.52]	−1.06 [−3.90; 1.78]	−0.75 [−2.79; 1.29]	−0.75 [−3.60; 2.10]	−0.13 [−2.81; 2.55]	Fluoxetine			
−9.85 [−24.87; 5.17]	−4.03 [−17.53; 9.47]	−1.11 [−3.96; 1.74]	−0.80 [−2.86; 1.26]	−0.80 [−3.66; 2.06]	−0.18 [−2.87; 2.52]	−0.05 [−2.95; 2.85]	Nutraceutical		
−10.77 [−25.69; 4.15]	−4.95 [−18.33; 8.43]	−2.03 [−4.52; 0.46]	**−1.72 [−3.23; −0.20]**	−1.72 [−4.22; 0.78]	−1.10 [−3.26; 1.07]	−0.97 [−3.51; 1.57]	−0.92 [−3.47; 1.64]	Lidocaine	
−10.74 [−25.60; 4.12]	−4.92 [−18.24; 8.40]	−2.00 [−4.13; 0.13]	**−1.69 [−2.48; −0.89]**	−1.69 [−3.83; 0.45]	−1.07 [−2.80; 0.67]	−0.94 [−3.13; 1.25]	−0.89 [−3.10; 1.32]	0.03 [−1.26; 1.32]	Sham/Placebo

Figure S3. Depression graph.

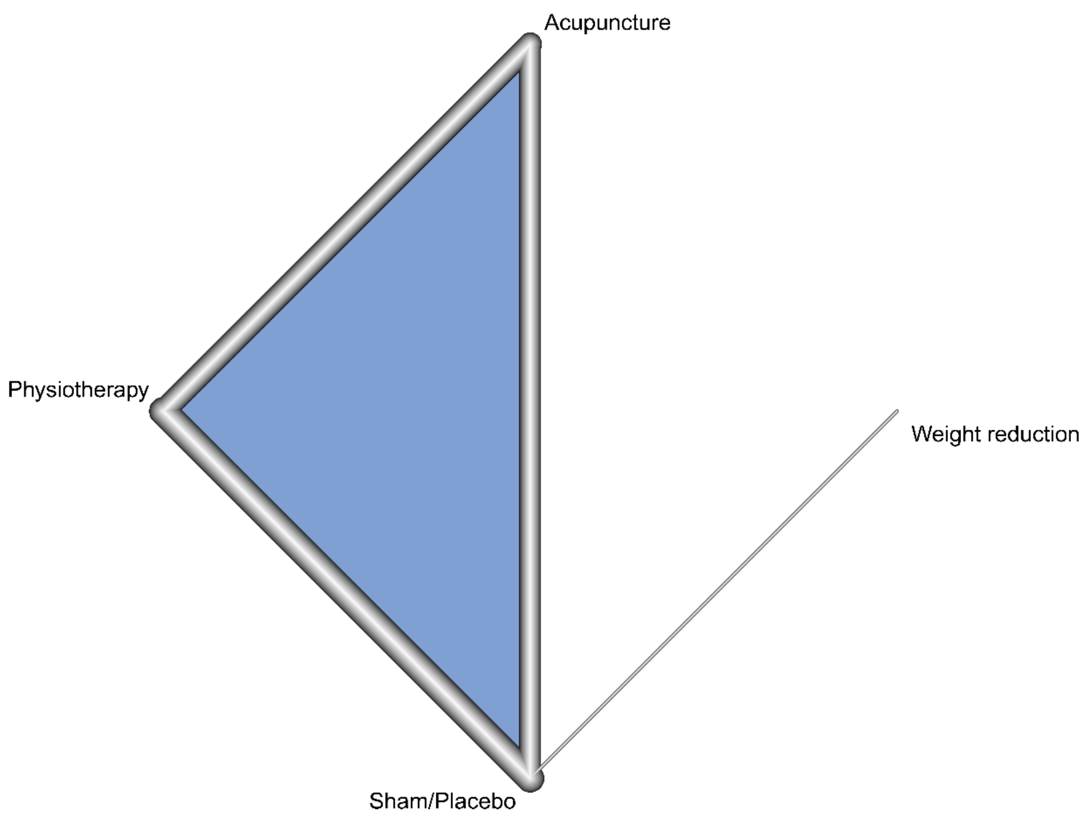

tau-squared = 39.7; I-squared = 99.6%; P = < 0.0001

Table S3. A network meta-analysis results regarding depression change.

Acupuncture			
−3.86 [−15.04; 7.32]	Physiotherapy		
−4.14 [−18.13; 9.85]	−0.28 [−16.97; 16.41]	Weight reduction	
**−9.64 [−16.13; −3.14]**	−5.78 [−16.96; 5.40]	−5.50 [−17.89; 6.89]	Sham/Placebo

Figure S4. Stiffness graph.

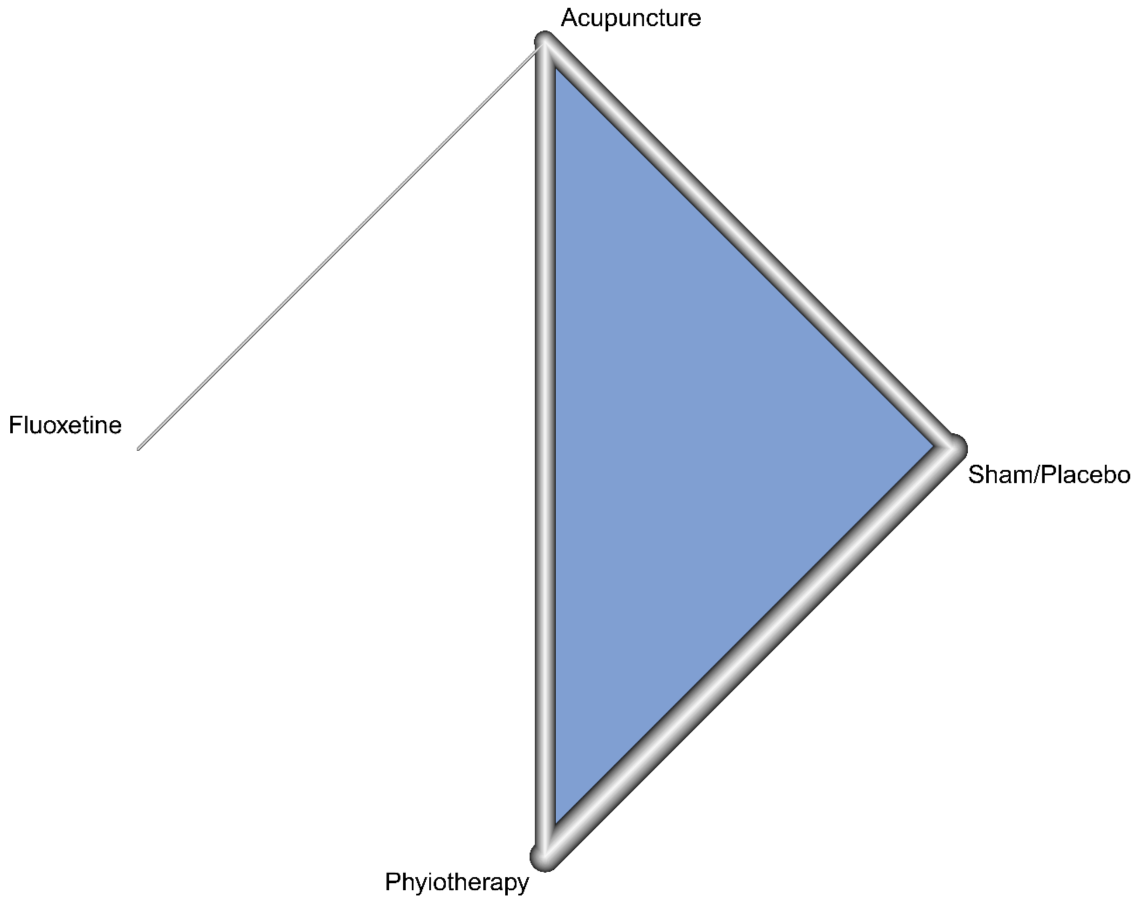

tau-squared = 102.5; I-squared = 95.3%; P < 0.0001

Table S4. A network meta-analysis results regarding stiffness change.

Acupuncture			
−2.00 [−21.85; 17.85]	Fluoxetine		
−3.87 [−22.06; 14.31]	−1.87 [−28.79; 25.04]	Physiotherapy	
−8.52 [−20.40; 3.36]	−6.52 [−29.65; 16.61]	−4.64 [−22.83; 13.54]	Sham/Placebo
